# Chaperone Activity and Protective Effect against Aβ-Induced Cytotoxicity of *Artocarpus camansi* Blanco and *Amaranthus dubius* Mart. ex Thell Seed Protein Extracts

**DOI:** 10.3390/ph16060820

**Published:** 2023-05-31

**Authors:** David Sanchez-Rodriguez, Idsa Gonzalez-Figueroa, Merlis P. Alvarez-Berríos

**Affiliations:** Department of Science and Technology, Inter American University of Puerto Rico at Ponce, Ponce, PR 00715-1602, USA; davsanch@iu.edu (D.S.-R.); igon0142@interponce.edu (I.G.-F.)

**Keywords:** Alzheimer’s disease, chaperones, protein extract, fibril inhibition, protective effect

## Abstract

Alzheimer’s disease (AD) is the most common type of dementia and is listed as the sixth-leading cause of death in the United States. Recent findings have linked AD to the aggregation of amyloid beta peptides (Aβ), a proteolytic fragment of 39–43 amino acid residues derived from the amyloid precursor protein. AD has no cure; thus, new therapies to stop the progression of this deadly disease are constantly being searched for. In recent years, chaperone-based medications from medicinal plants have gained significant interest as an anti-AD therapy. Chaperones are responsible for maintaining the three-dimensional shape of proteins and play an important role against neurotoxicity induced by the aggregation of misfolded proteins. Therefore, we hypothesized that proteins extracted from the seeds of *Artocarpus camansi* Blanco (*A. camansi*) and *Amaranthus dubius* Mart. ex Thell (*A. dubius*) could possess chaperone activity and consequently may exhibit a protective effect against Aβ_1–40_-induced cytotoxicity. To test this hypothesis, the chaperone activity of these protein extracts was measured using the enzymatic reaction of citrate synthase (CS) under stress conditions. Then, their ability to inhibit the aggregation of Aβ_1–40_ using a thioflavin T (ThT) fluorescence assay and DLS measurements was determined. Finally, the neuroprotective effect against Aβ_1–40_ in SH-SY5Y neuroblastoma cells was evaluated. Our results demonstrated that *A. camansi* and *A. dubius* protein extracts exhibited chaperone activity and inhibited Aβ_1–40_ fibril formation, with *A. dubius* showing the highest chaperone activity and inhibition at the concentration assessed. Additionally, both protein extracts showed neuroprotective effects against Aβ_1–40_-induced toxicity. Overall, our data demonstrated that the plant-based proteins studied in this research work can effectively overcome one of the most important characteristics of AD.

## 1. Introduction

Alzheimer’s disease (AD) is considered the most common type of dementia and is listed as the sixth-leading cause of death in the United States [1]. Currently, 6.7 million Americans aged 65 and older are living with AD and it is expected that global AD patients will triple by 2050 [2,3]. AD is characterized by the formation of amyloid-beta (Aβ) peptide aggregates on senile plaques (SP) and neurofibrillary tangles formed by tau deposition [3,4,5]. Amyloid-beta (Aβ) peptide is a proteolytic fragment of 39–43 amino acid residues derived from the amyloid precursor protein (APP). SP are mainly composed of the 40- (Aβ_1–40_) and 42-residue-long peptides (Aβ_1–42_), where Aβ_1–40_ is the most abundant and Aβ_1–42_ the more aggregation-prone [6]. The aggregation of Aβ_1–40_ and Aβ_1–42_ into insoluble fibrils is considered the major pathological hallmark of AD [3,7,8]. It has proven to be toxic, leading to memory loss and cognitive decline over time. The amyloid cascade hypothesis of AD proposes that this aggregation is the primary event that ultimately leads to AD dementia [9].

Currently, no preventive or effective treatment for AD is available. Acetylcholinesterase inhibitors and N-methyl-D-aspartate receptor antagonists are the standard drugs for the treatment of AD and only provide temporary alleviation of the psychological and behavioral symptoms [10,11]. Therefore, new therapies to inhibit the aggregation or removal of amyloid-beta (Aβ) peptides are currently being explored as promising strategies for AD treatment [8,10,12,13,14,15]. For example, in 2021 the FDA approved a new drug (Aducanumab) through the accelerated approval program that removes amyloid deposits in the brain. Although this drug is a promising treatment, it induces severe side effects such as brain swelling or bleeding in the brain [16]. 

In recent decades, plant-based medications have gained significant attention for the treatment of neurological disorders [17,18,19]. The bioactive constituents of plants can exhibit therapeutic activity against AD, providing a new avenue for the discovery of drugs with minimal side effects. Studies have demonstrated the efficacy of different natural compounds of plant origin such as alkaloids, terpenoids and phenolic compounds against the aggregation of Aβ [17,20,21,22,23,24,25]. Bastianetto et al. [26] reported that the flavonoids present in the leaf extract of the *Ginkgo biloba* L. tree may be able to protect hippocampal cells against toxic effects induced by Aβ peptides. Extracts from *Allium roseum* L., a plant rich in organosulfur compounds and flavonoids, inhibited the fibrilization process of Aβ_1–42_ and provided a neuroprotective effect to SH-SY5Y human neuroblastoma cells by hindering the formation of mature fibrils [27]. Similarly, *Lawsonia inermis* L., *Punica granatum* L. and *Pistacia lentiscus* L. extracts inhibited the aggregation and induced neurotoxicity of Aβ_1–42_. Although these natural compounds have demonstrated in vitro and in vivo efficacy against this deadly disease, plants may also contain other bioactive molecules with superior anti-AD activity [28]. 

Several reports have revealed that chaperones and chaperone-like proteins have the ability to interfere with amyloid beta formation, preventing the toxicity that is associated with this process [29,30,31,32,33,34]. Chaperones play a key role in helping other proteins to maintain their correct shapes, preventing aggregation and its potentially cytotoxic effects [35]. Hochberga et al. [36] established that the chaperone αB-crystallin prevented Aβ fibril formation and consequently reduced its toxicity in HEK293, HeLa and PC12 cells. Arosio et al. [37] demonstrated that the molecular chaperones DNAJB6, Ssa1 and proSP-C Brichos were able to exert a protective function against the aggregation of Aβ_1–42_ through diverse mechanisms. Although the use of chaperones has shown promising results, they are highly toxic or reveal a lack of specificity [38]. Therefore, the exploration of plant-based proteins, especially those with chaperone activity, may be a key strategy to overcome one of the main hallmarks of AD. 

Recent research has reported that Artocarpus and Amaranthus species contain potential bioactive molecules with pharmacological properties, such as antioxidant, anti-inflammatory, antibacterial and anticarcinogenic activities [39,40]. *Artocarpus camansi* Blanco (*A. camansi*) and *Amaranthus dubius* Mart. ex Thell (*A. dubius*), belonging to the Artocarpus and Amaranthus genera, respectively, are mainly distributed in the tropical regions, including Puerto Rico. These species have demonstrated remarkable anticancer and antioxidant properties [41,42]. To our knowledge, the chaperone activity and the neuroprotective effects of these plants against Aβ toxicity have not been evaluated in vitro. Seed plants are rich in proteins that are known to act as chaperones, such as heat shock proteins and the late embryogenesis abundant proteins [43,44]. Thus, in this study, the chaperone activity of seed protein extracts obtained from *A. dubius* and *A. camansi* was investigated. We hypothesize that *A. camansi* and *A. dubius* seed protein extracts may inhibit Aβ_1–40_ fibrils and provide a protective effect to SH-SY5Y human neuroblastoma cells due to their chaperone activity. To test this hypothesis, the enzymatic reaction of citrate synthase was used to determine the chaperone activity of each protein extract. Thioflavin T and DLS measurements were performed to test the ability of the extracts to inhibit the formation of Aβ_1–40_. In addition, neuroprotective studies using SH-SY5Y cells were carried out. Our results indicated that *A. camansi* and *A. dubius* seed protein extracts showed chaperone activity and were able to inhibit Aβ_1–40_ fibrillization, with *A. dubius* showing the highest efficacy. Moreover, they were able to provide protection to SH-SY5Y cells against Aβ_1–40_-induced toxicity. This neuroprotective effect may be attributed to the chaperone activity of the proteins present in the extracts, which inhibited the formation of toxic Aβ_1–40_ fibrils.

## 2. Results and Discussion

### 2.1. Preparation of Protein Extracts and Protein Concentration

Plants may provide a plethora of new therapeutic drugs for the treatment of AD [17,18,26]. In this study, seed protein extracts were obtained from *A. camansi* and *A. dubius* with the intention to evaluate their chaperone activity and in vitro therapeutic potential as anti-AD therapy. Proteins were extracted in 50 mM PBS (pH 7.4) and partially purified using ammonium sulfate precipitation and dialysis. The total protein concentrations of the *A. camansi* and *A. dubius* protein extracts were 6043.2 μg/mL and 675.7 μg/mL, respectively, as determined by the bicinchoninic acid (BCA) assay. 

*A. camansi*, a type of plant (tree) that belongs to the family Moraceae [45], has larger seeds than *A. dubius*, a type of plant (leafy vegetable) from the family Amaranthaceae [46]. Large seed size has been shown to be associated with length of life cycle and habitat stability, while smaller seeds are characteristic of parasites that are species of plants that form seed banks and have higher distribution [47]. Having a large seed provides higher survivability for plants against drought and isolation on remote islands. This indicates that larger seeds have more resources towards survivability; hence, this could explain the notable protein concentration difference between the two protein extracts. Several studies have shown that *A. camansi* protein content ranges from 4.87 to 19.6% (dry weight), with high variability due to the plant’s location [48]. On the other hand, there is no information reported in the literature regarding the protein content of *A. dubius* seeds. To compare the potential inhibitory efficacy of both plant extracts against Aβ_1–40_ fibril formation under the same conditions, *A. camansi* and *A. dubius* seed protein extracts were used at the same concentration for further experiments. 

### 2.2. Protein Profiling with SDS-PAGE

**Figure 1:**
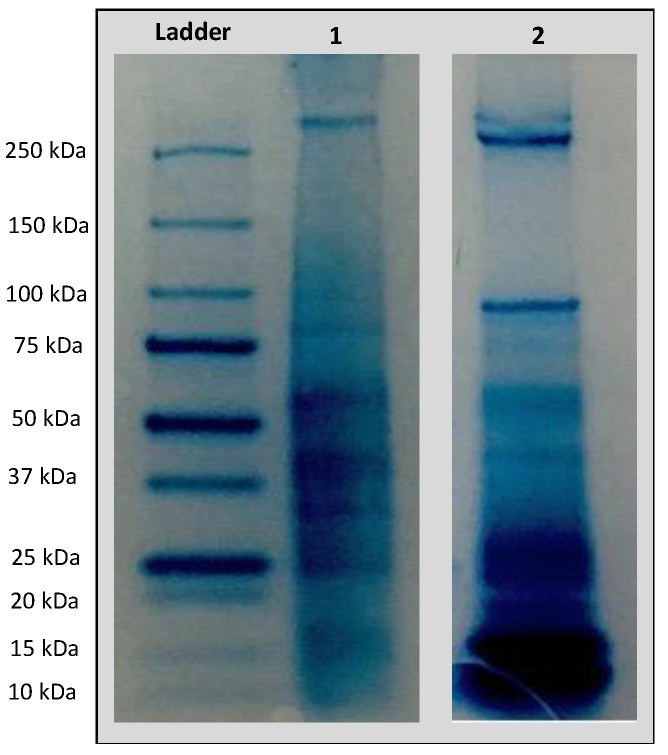
SDS-PAGE analysis of *A. dubius* (lane 1) and *A. camansi* (lane 2) protein extracts.

SDS-PAGE electrophoresis is widely used to determine the electrophoretic profile of representative samples including different species of plants [49]. To estimate the protein pattern of protein extracts obtained from *A. dubius* and *A. camansi*, SDS-PAGE electrophoresis was carried out. The results suggested that both extracts contained proteins with diverse molecular weight distributions (Figure 1). *A. dubius* protein bands ranged from approximately 75 kDa to 15 kDa, and there was a band above 250 kDa. Similarly, *A. camansi* bands ranged from approximately 100 kDa to 10 kDa. A band above 250 kDa was also observed. 

### 2.3. CD Spectra and Estimation of Protein Secondary Structure Content 

**Figure 2:**
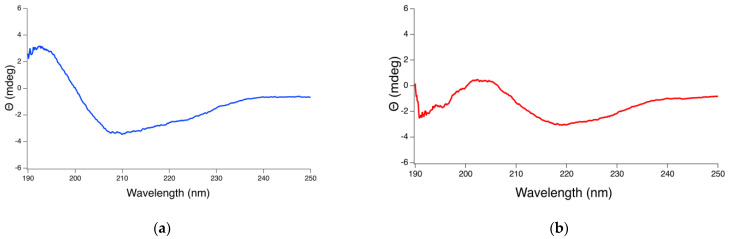
CD spectra of seed protein extracts obtained from (**a**) *A. dubius* and (**b**) *A. camansi*.

To obtain information about the secondary structure of the proteins present in the seed extracts, CD spectra were recorded and analyzed using the server BeStSel [50]. CD spectra for *A. dubius* protein extracts indicated a positive maximum at 196, suggesting the presence of β-sheet conformation, and a negative minimum at 208 nm, characteristic of α-Helix conformations (Figure 2). *A. camansi* spectra showed a negative minimum at 218 assigned to the β-sheet structure [51,52]. 

Secondary structure analysis using the BeStSel method suggested that the *A. dubius* protein extract consisted of 5.9% of α-helix, 25.6% of β-Sheet, 13.5% of β-Turn and 54.9% of “others” (Table 1). The protein extract obtained from *A. camansi* consisted of 36.8% of β-Sheet, 12.4% of β-Turn and 50.7% of “others”. Others, which include 3_10_-helix, π-helix, β-bridge, bend, loop/irregular and invisible regions of the structure, accounted for the majority of the secondary structures in the *A. camansi* and *A. dubius* protein extracts.

### 2.4. Chaperone Activity of Seed Protein Extracts of A. camansi and A. dubius

**Figure 3:**
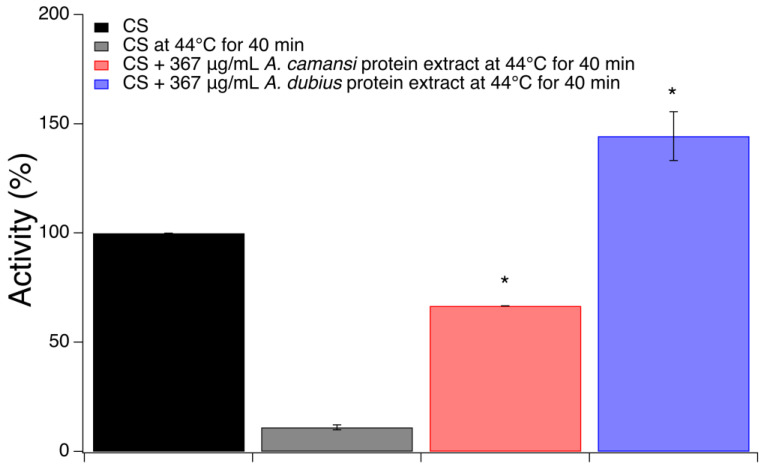
Chaperone activity of *A. camansi* or *A. dubius* protein extracts by evaluating their effect on the activity of CS under high-temperature stress conditions (44 °C for 40 min). The error bars represent the standard error of three independent experiments. * Statistical significance (*p* < 0.05) between *A. camansi* or *A. dubius* protein extract and CS alone at 44 °C for 40 min.

Among plant bioactive molecules, proteins with chaperone activity are a promising anti-AD strategy due to their ability to inhibit the aggregation of Aβ peptide [29,30,34]. To elucidate if *A. camansi* and *A. dubius* protein extracts possess such a function, the enzymatic reaction of citrate synthase (CS) was used to measure their chaperone activity. The chaperone activity of a protein is determined by the protection of a client protein against loss of activity under stress conditions [53]. Therefore, the activity of CS in the presence or absence of *A. camansi* or *A. dubius* protein extracts at 44 °C for 40 min was determined. Activity of 100% was set to be the activity of CS without protein extracts before stress conditions. The results showed that the activity of CS decreased from 100% (before stress) to 11% (after stress) in the absence of the protein extracts (Figure 3). This result is consistent with previous reports where the activity of CS drops from 100% to under 20% in these conditions [53]. The protective effect of both protein extracts was evident when CS was exposed to temperature stress at the concentration assessed. The presence of *A. camansi* protein extract resulted in a significant increase in CS activity when compared with CS alone under stress conditions (66.7 ± 0.0% activity for CS + *A. camansi* and 11.1 ± 1.1% of activity for CS alone, *p* < 0.05). The enzymatic activity of CS is completely protected against temperature stress by the presence of *A. dubius* (144.4 ± 11.1% for CS + *A. dubius* compared with 11.1 ± 1.1% for CS alone, *p* < 0.05). The apparent activity of CS above 100% is a phenomenon that has been observed in several experiments with chaperones. It is believed that the reactivation of inactive species in the commercial enzyme product is mainly responsible for this increase [53,54]. It was observed that *A. dubius* protein extract can protect CS against temperature inactivation to a higher extent than *A. camansi* protein extract, suggesting that the concentration of proteins with chaperone activity is higher in *A dubius*. These results let us speculate that the two seed protein extracts studied in this work may possess potent inhibitory activity against Aβ fibril formation and a neuroprotective effect against Aβ-induced toxicity. 

### 2.5. Inhibition of Aβ_1–40_ Fibrillation Using ThT Assay

**Figure 4:**
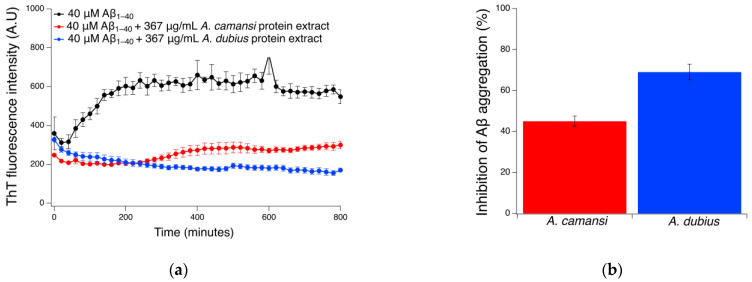
(**a**) Thioflavin T fluorescence intensity as a function of time for 40 μM Aβ_1–40_ (black), in the presence of 367 μg/mL of seed protein extracts obtained from *A. camansi* (red) or *A. dubius* (blue). (**b**) Inhibition of Aβ_1–40_ aggregation in the presence of *A. camansi* or *A. dubius* seed protein extracts. The error bars represent the standard error of three independent experiments.

The formation and accumulation of beta amyloid fibrils is one of the distinctive characteristic events in Alzheimer’s pathophysiology [55]. The inhibition effect of *A. dubius* and *A. camansis* protein extracts against Aβ_1–40_ fibril formation was examined using the fluorescent probe Thioflavin T at 37 °C. Thioflavin T is a fluorescent dye that exhibits enhanced fluorescence in the presence of amyloid fibrils, allowing it to act as an indicator for evaluating beta amyloid fibril formation and inhibition [56]. An increase in fluorescence intensity would be indicative of the presence of fibrils, while a decrease is indicative of inhibition. The results demonstrated that the seed protein extracts from *A. dubius* and *A. camansi* were effective in inhibiting Aβ_1–40_ fibrilization, with *A. dubius* extract showing the highest inhibition (Figure 4a). The fluorescence intensity of the control sample (Aβ_1–40_) occurred parabolically until reaching a plateau at 180 min (3 h), indicating the formation of amyloid beta fibrils. This trend is consistent with the literature, where amyloid fibril concentration increases in a parabolic manner until reaching a plateau [57,58]. The presence of protein extract from *A. camansi* demonstrated a 44% reduction in fluorescence intensity when compared with the untreated control (Figure 4a). On the other hand, the presence of *A. dubius* seed protein extract (Figure 4) demonstrated an 82% reduction in ThT fluorescence intensity when compared with control samples. After co-incubating Aβ_1–40_ peptide with the protein extracts for 13 h, the *A. dubius* and *A. camansi* protein extracts showed 69.0 ± 3.8% and 45.3 ± 2.5% inhibition of Aβ_1–40_ aggregation, respectively (Figure 4b). These results suggest that the high chaperone activity of *A. dubius* seed protein extract as shown in Figure 3 may be responsible for its superior inhibitory effect on Aβ_1–40_ aggregation at the concentration assessed. 

### 2.6. Size Distribution Analysis with DLS 

Dynamic light scattering (DLS) has been used to determine the size distribution of Aβ_1–40_ aggregates [59]. It provides information about the size for all molecules in a solution [60]. To further confirm the Aβ_1–40_ fibril inhibitory capacity of the seed protein extracts, the size distribution of Aβ_1–40_ incubated in the presence or absence of the *A. camansis* or *A. dubius* seed protein extracts was determined. Large diameters are considered as an indication of the presence and/or formation of amyloid fibrils and small diameters indicate a reduction in amyloid fibrillogenesis. 

**Figure 5:**
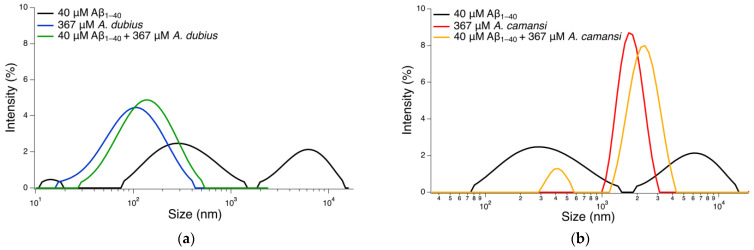
Overlay of size distribution by intensity for untreated Aβ_1–40_ fibrils and Aβ_1–40_ fibrils treated with (**a**) *A. dubius* protein extract or (**b**) *A. camansi* protein extract.

Untreated Aβ_1–40_ fibrils showed peaks at ~7048 nm and 332 nm (Figure 5). When Aβ_1–40_ was co-incubated with *A. dubius* protein extract, a small shift in the peak characteristic for *A. dubius* protein extract alone from ~103 nm to ~153 nm was observed (Figure 5a). An increasing number of reports have demonstrated that chaperones or proteins with chaperone activity can bind to oligomeric species of Aβ, interfering with the process of fibril formation [61,62,63]. Therefore, this peak shift may be attributed to the interaction of the Aβ_1–40_ peptides/oligomers with *A. dubius* proteins present in the solution, providing these proteins with the ability to inhibit the formation of small and large Aβ_1–40_ aggregates. The *A. camansi*-treated sample showed a shift in the peak corresponding to *A. camansi* alone from ~1821 to ~2322, also demonstrating its capacity to interact with Aβ_1–40_ peptide/oligomers (Figure 5b). An additional small peak at 450 nm was observed, indicating the ability of *A. camansi* extract to inhibit only the formation of large Aβ_1–40_ aggregates. 

The DLS results were consistent with those obtained from ThT studies where both protein extracts demonstrated inhibitory properties towards Aβ_1–40_ fibrillogenesis, with *A. dubius* showing the highest inhibition efficacy. It has been reported that the inhibitory capacity of chaperones against amyloid beta fibril formation is concentration-dependent [64]. As displayed in Figure 3, *A. dubius* exhibited the highest chaperone activity; hence, this potent inhibitory effect may be due to the high concentration of chaperones in this protein extract. To our knowledge, there are no specific articles that address the inhibitory properties of *A. dubius* and *A. camansi* towards beta amyloid fibril formation. For the first time, we showed that *A. dubius* and *A. camansi* seed protein extracts are promising inhibitors of the Aβ fibrillation process, one of the main hallmarks of AD. 

### 2.7. Hydrolysis of Seed Protein Extracts Using the Alcalase Enzyme

**Figure 6:**
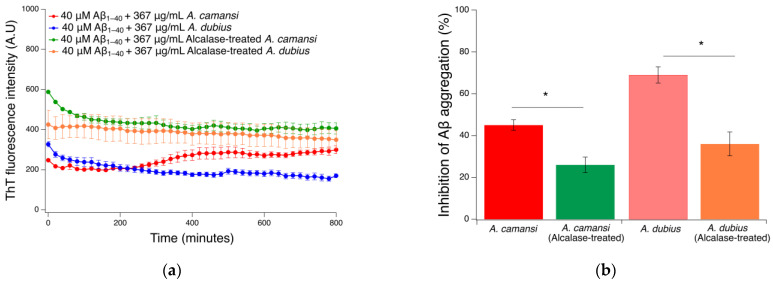
(**a**) ThT fluorescence intensity as a function of time for 40 μM Aβ_1–40_ in the presence of 367 μg/mL of untreated or alcalase-treated seed protein extracts obtained from *A. camansi* or *A. dubius*. (**b**) Inhibition of Aβ_1–40_ aggregation in the presence of protein extracts treated or untreated with alcalase. The inhibition percentage of Aβ_1–40_ aggregation was calculated using the ThT fluorescence intensities of the control sample (Aβ_1–40_) and Aβ_1–40_ in the presence of treated or untreated seed protein extracts using Equation (1). Data are represented as the mean ± SE of four independent experiments. * *p* < 0.05 (Student’s *t*-test).

To confirm that proteins with chaperone activity present in the *A. camansi* and *A. dubius* seed extracts were responsible for inhibiting the fibrillization process of Aβ_1–40_ as demonstrated by the ThT and DLS results, the protein extracts were incubated with the proteolytic enzyme alcalase, which provides very extensive hydrolysis of plant proteins. If these proteins played a key role in fibril inhibition, the co-incubation of Aβ_1–40_ with alcalase-treated protein extracts would result in an increase in ThT fluorescence intensity (increase in amyloid fibril concentration) when compared with untreated seed protein extracts. The results showed that the protein breakdown induced by alcalase decreased the Aβ_1–40_ fibril inhibition activity of both extracts (Figure 6a). Increases of ~35% and ~100% in ThT fluorescence intensity were observed in the alcalase-treated *A. camansi* and *A. dubius* seed protein extract, respectively, when compared with untreated samples. This increase in fluorescence intensities may be attributed to the hydrolysis of key proteins with chaperone activity in the *A. camansi* and *A. dubius* samples responsible for fibril inhibition. After 13 h of incubation with the alcalase-treated protein extracts, the inhibition of Aβ_1–40_ aggregation decreased considerably when compared with the untreaded protein extracts, as shown in Figure 6b (*A. camasi* protein extract: 45.3 ± 2.5% for untreated sample and 25.9 ± 3.7% for alcalase-treated samples, *p* < 0.05; *A. dubius* protein extract: 69.0 ± 3.8% for untreated sample and 36.2 ± 5.7% for alcalase-treated sample, *p* < 0.05). Overall, our findings confirmed that the proteins that were hydrolyzed within the samples are important for the inhibition of Aβ_1–40_ fibril formation. 

### 2.8. Cytotoxic Effect of Protein Extracts on SH-SY5Y Cells

**Figure 7:**
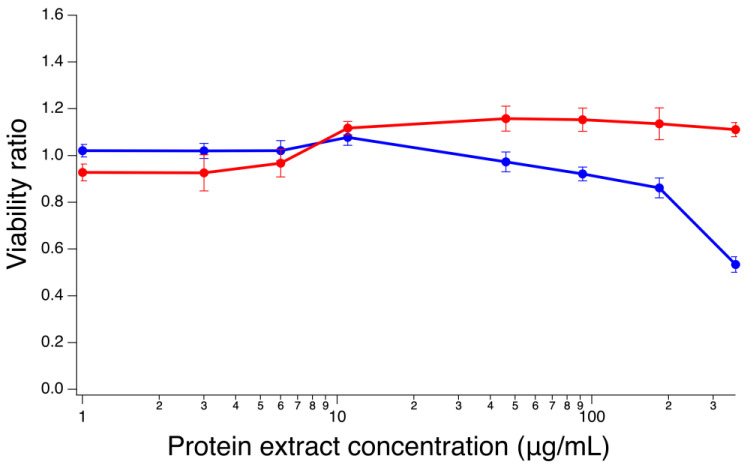
Dose–response curve for SH-SY5Y cells. Cells were exposed to varying concentrations of *A. camansi* (red) or *A. dubius* (blue) protein extracts (1–367 μg/mL) for 48 h. Error bars represent the standard error of the mean of three independent experiments.

To determine whether *A. camansi* and *A. dubius* seed protein extracts are cytotoxic, SH-SY5Y neuroblastoma cells were exposed to increasing concentrations of protein extracts for 48 h. Following this incubation period, cells were assayed for viability using a CellTiter 96^®^ aqueous assay. The protein extract from *A. camansi* was not toxic to SH-SY5Y cells in the range assessed (1 μg/mL, 3 μg/mL, 6 μg/mL, 11 μg/mL, 46 μg/mL, 92 μg/mL, 184 μg/mL and 367 μg/mL). In contrast, the toxicity of the *A. dubius* protein extract was dose-dependent, and the viability of cells decreased at higher concentrations of the seed protein extract (92 μg/mL, 184 μg/mL and 367 μg/mL) (Figure 7). For neuroprotective studies, nontoxic protein extract concentrations were chosen (6 μg/mL, 11 μg/mL and 46 μg/mL). 

### 2.9. Neuroprotective Effect of A. camansi and A. dubius Protein Extracts in SH-SY5Y Cells

**Figure 8:**
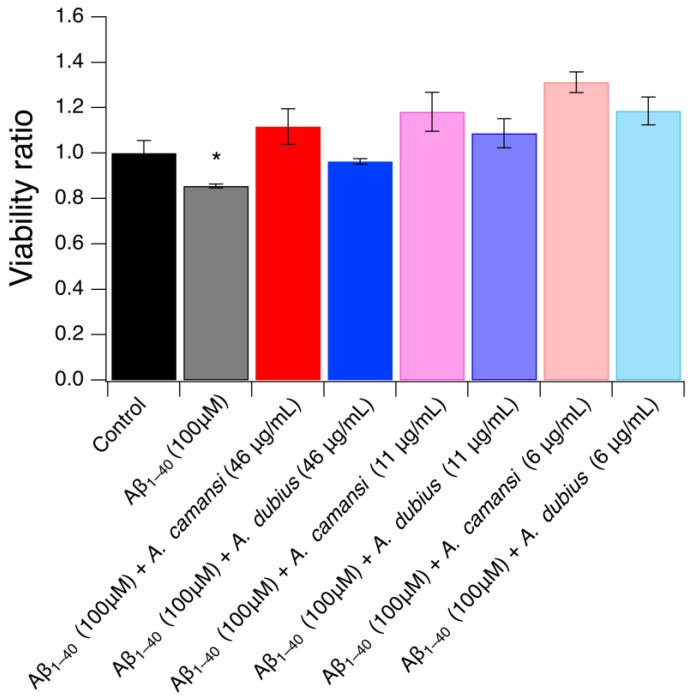
Evaluation of the protective effect of *A. camansi* or *A. dubius* seed protein extracts against cytotoxicity induced by Aβ_1–40_ in SH-SY5Y cells. The viability ratio was determined after incubating SH-S5Y5 cells with Aβ_1–40_ fibrils in the presence or absence of *A. camansi* or *A. dubius* protein extracts for 48 h. Error bars represent the standard deviation of the mean of three independent experiments. * Statistical significance (*p* < 0.05) between Aβ_1–40_-treated cells and co-incubation of Aβ_1–40_ with *A. camansi* or *A. dubius* protein extracts.

To explore the promising neuroprotective effect of the *A. camansi* and *A. dubius* seed protein extracts against Aβ_1–40_ fibrils, Aβ_1–4__0_ (100 μM) was incubated at 37 °C in the presence or absence of protein extracts at the nontoxic concentrations of 46 μg/mL, 11 μg/mL or 6 μg/mL for 24 h. Afterwards, SH-SY5Y cells were co-incubated with these solutions for 48 h and the viability ratio was evaluated using the CellTiter 96^®^ aqueous assay. The results revealed that Aβ_1–4__0_ fibrils at a concentration of 100 μM showed a significant decrease in cell viability relative to untreated cells (*p* < 0.05) (Figure 8). However, the co-incubation of Aβ_1–40_ with *A. camansi* or *A. dubius* protein extracts mitigated the cell mortality induced by Aβ_1–40_ amyloid fibrils at the three concentrations assessed. A statistically significant difference (*p* < 0.05) between the viability ratio of cells incubated with Aβ_1–40_ alone and cells incubated in the presence of the protein extracts was observed. This neuroprotective effect may be due to the chaperone activity of the proteins present in the extracts that were able to inhibit the formation of toxic Aβ_1–40_ fibrils. Overall, our results indicated that both protein extracts are a promising anti-AD therapy in the conditions tested. 

## 3. Materials and Methods

### 3.1. Materials

Phosphate-buffered saline (PBS), ammonium sulphate, alcalase, citrate synthase from porcine heart, oxaloacetate, acetyl coenzyme A sodium salt, 5,5′-Dithiobis(2-nitrobenzoic acid) (DTNB), Tris-HCl buffer, thioflavin T (ThT) and all the materials for cell culture were purchased from Sigma-Aldrich (St Louis, MO, USA). Bicinchoninic acid (BCA) assay kit was obtained from Fisher Scientific (Hampton, NH, USA). Aβ_1–40_ was purchased from Anaspec (San Jose, CA, USA). Bio-Safe™ Coomassie stain, precision plus protein kaleidoscope standards and TGX precast gels were purchased from Bio-Rad (Hercules, CA, USA).

### 3.2. Plant Material

*A. dubius* and *A. camansi* were naturally cultivated and obtained from a local farm in Yauco, PR. Plants were identified by a local expert named David Sanchez Montalvo who has extensive knowledge in folkloric medicine of endemic and nonendemic plants found in Puerto Rico. The local names and aerial parts used of each selected plant are shown in Table 2. 

### 3.3. Protein Extraction and Purification

Protein extraction and purification were performed using a method previously reported in the literature with some modifications [65]. Briefly, the two different types of seeds from Puerto Rican plants (Table 2) were collected in Yauco, PR, sun dried for 20 h and dried further using a lyophilizer. The seed of each plant was pulverized using a mortar and pestle and mixed with 50 mM PBS buffer (pH 7.4) using a 1:9 ratio (1 g of seed in 9 mL of buffer). Then, the extracts were centrifuged at 10,000 rpm at 4 °C for 20 min. After centrifugation, the protein crude extract (supernatant) was transferred to a fresh Eppendorf tube and saturated with ammonium sulphate until 80% saturation was achieved. The saturated crude extract was centrifuged at 12,000 rpm and 4 °C for 40 min, the supernatant was carefully discarded, and the pellet was resuspended in 50 mM PBS buffer and dialyzed using a Pur-A-Lyzer™ Maxi dialysis tube. The dialysis tube was first submerged into the 50 mM PBS buffer for 10 min. Afterwards, the protein solution was poured into the dialysis tube and placed in a beaker filled with 50 mM PBS buffer under continuous stirring. The buffer was changed three times every three hours. After the dialysis process, the protein solution was stored in small aliquots at −20 °C until use. The BCA protein assay kit was used to determine the concentration of protein in each seed extract [66]. 

### 3.4. SDS-PAGE Pattern of Seed Protein Extracts

The protein profile of the crude seed protein extracts was analyzed using sodium dodecyl sulfate polyacrylamide gel electrophoresis (SDS PAGE) according to the procedure described by Soto-Madrid et al. [67] with some modifications. For this, each seed protein extract (12 μg for *A. dubius* and 23 μg for *A. camansi*) was mixed with 8 μL of Laemmli buffer and incubated for 5 min at 95 °C. Samples and the prestained protein standard were loaded into TGX precast gels and run at 200 volts for 25 min in the Mini-PROTEAN Tetra cell electrophoresis module (Bio-Rad, Hercules, CA, USA). Afterwards, gels were stained with Bio-Safe™ Coomassie stain for two hours, and left destaining overnight.

### 3.5. Circular Dichroism Spectroscopy

Circular dichroism (CD) measurements of the protein extracts were collected using a J-1100 spectrophotometer (Jasco Inc., Tokyo, Japan) and a 1 mm path length cuvette thermostated at 20 °C. Each spectrum was measured from 190 to 250 nm, with six scans at a scanning speed of 200 nm/min, a 1.00 nm bandwidth and a spectral resolution of 0.1 nm. The CD spectra of the solvent were recorded and subtracted from the sample’s spectra. To estimate the major secondary conformations of the proteins present in the seed extracts, the server BeStSel was used [50]. 

### 3.6. Chaperone Activity Determination

Chaperone activity of the seed protein extracts was measured using a protocol developed by Hristozova et al. [53]. The protocol fits the format of a microplate reader and is based on the enzymatic reaction of citrate synthase (CS) and the ability of chaperones to protect its enzymatic activity under stress conditions (heat). First, the enzymatic activity of CS was evaluated before it was subjected to stress (heat). After this, 100 μL of a reaction solution containing 6 nM of CS, 0.45 mM of acetyl-coA, 0.5 mM of oxaloacetate and 0.1 mM of DTNB in mM50 mM Tris-HCl Buffer (pH 7.5) was followed for three minutes (samples were shaken and measurements were recorded every 20 s) at 30 °C and 412 nm using a TECAN Infinite M Plex microplate reader. To study the protective effect of the protein extracts against thermal deactivation of the CS, a reaction mixture containing CS and seed protein extracts in 50 mM Tris-HCl buffer at pH 7.5 was added to a 96-well plate and incubated at 44 °C for 40 min. After this period, acetyl-coA, oxaloacetate and DTNB were added to each heat-treated sample and immediately measured at 20 s intervals for three minutes with orbital shaking before each measurement using a TECAN Infinite M Plex microplate reader. Acetyl-coA, oxaloacetate and DTNB were added just before measurements to avoid thermal decomposition. The final volume of each well was 100 μL and contained the following final concentrations: CS (6 nM), acetyl-coA (0.45 mM), oxaloacetate (0.5 mM), DTNB (0.1 mM) and protein extracts (367 μg/mL). The absorbance of a sample blank for each extract (100 μL of protein extract at 367 μg/mL in 50 mM Tris-HCl buffer) was measured and subtracted from the sample’s measurements. The activity of CS was determined using the slope of the initial, linear phase of the curve [53]. Relative activity of the samples was calculated by determining the ratio between the activity of each sample and the activity of the enzyme before it was exposed to stress. 

### 3.7. Thioflavin T Fluorescence Measurements 

Thioflavin T is a benzothiazole dye that exhibits enhanced fluorescence upon binding to amyloid fibrils and is widely used to monitor their formation [56]. To evaluate the inhibition of Aβ_1–40_ fibrils by *A. dubius* and *A. camansi* seed protein extracts, thioflavin T studies were performed. In accordance with Sudhakar et al. [57], 1 mg of Aβ_1–40_ peptide was dissolved in 40 μL of a NH_4_OH solution at room temperature. Then, 960 μL of a 50 mM PBS buffer was added to obtain a final Aβ_1–40_ peptide concentration of 1 mg/mL (235.9 μM). A solution of ThT was prepared in 50 mM PBS (35 μM). Then, reaction mixtures with a final volume of 140 μL were prepared in a 96-well plate. Each well contained the following final concentrations: ThT (10 μM), Aβ_1–40_ peptide (40 μM) and protein extracts (367 μg/mL). Controls (Aβ_1–40_ in the absence of protein extracts) contained 50 mM PBS (pH 7.4) instead of the protein extracts. The fluorescence intensities of the reaction mixtures as a function of time were measured at 37 °C and at excitation and emission wavelengths of 446 and 490 nm, respectively, using a TECAN Infinite M Plex microplate reader. The plate was gently shaken before each measurement. The following equation was used to determine the percentage inhibition of Aβ_1–40_ aggregation [68]:

(1) $$\left(1-\frac{Fa}{Fb}\right)\times 100$$

where *Fb* is the fluorescence intensity of Aβ_1–40_ in the absence of the protein extracts and *Fa* is the fluorescence intensity of Aβ_1–40_ in the presence of the protein extracts. 

### 3.8. DLS Measurements of Aβ_1–40_ Fibrils in the Presence or Absence of Seed Protein Extracts

Because beta amyloid fibrils are clusters or aggregations of the beta amyloid peptide, it is expected that these molecules have an increased diameter [69]. Therefore, if the seed protein extracts have the capability to inhibit Aβ_1–40_ fibril formation in vitro, the diameter exhibited by the amyloid beta peptide in the presence of the plant extracts should be considerably lower when compared with the diameter in the absence of the extracts. For this experiment, 1 mg/mL Aβ_1–40_ peptide solution was prepared as described in Section 3.7. Then, the reaction mixtures were prepared in PBS and microcentrifuge tubes containing the following final concentrations: Aβ_1–40_ peptide (40 μM) and protein extracts (367 μg/mL). The final volume in each microcentrifuge tube was 140 μL. Samples were incubated for 20 h at 37 °C and DLS measurements were performed using a particle size analyzer (NanoPlus HD, Micromeritics Instrument Corporation, Norcross, GA, USA).

### 3.9. Alcalase Hydrolysis of Seed Protein Extracts 

To evaluate if the proteins present in the extracts are the main ones responsible for inhibiting Aβ_1–40_ fibril formation, they were treated with the proteolytic enzyme alcalase, an endo-protease that allows the extensive hydrolysis of plant proteins [70]. This procedure was carried out in accordance with Parekh et al. [65] with some modifications. Alcalase was added to 120 μL of each protein extract using a 1:3 ratio. This mixture was incubated at 37 °C for 20 h. After treatment, the supernatant was recovered using centrifugation at 10,000 rpm for 5 min and used immediately for ThT fluorescence assay following the procedure established in Section 3.7.

### 3.10. Cell Culture

The neuroblastoma cell line SH-SY5Y, which has been used widely for neurotoxicity and neuroprotection studies, was purchased from Sigma-Aldrich. SH-SY5Y cells were cultured in 1:1 mixture of Eagle’s Minimum Essential Medium and Ham’s F12 medium supplemented with 15% FBS, 2mM L-glutamine, 1% nonessential amino acids, 1% streptomycin and 1% penicillin at 37 °C with 5% CO_2_ atmosphere. The culture media was changed every 2–3 days. The cells were used between passages 1 and 8 for all assays. All cell cultures were maintained in 75 cm^2^ cell culture flasks and the cells were passaged at 70–80% confluency every 3–5 days.

### 3.11. Cytotoxicity Studies of Seed Protein Extracts

A total of 12,500 cells/well were seeded in 96-well plates and allowed to adhere for 24 h at 37 °C with 5% CO_2_ atmosphere. After this period of time, the cell culture media was removed and cells were exposed to varying concentrations of *A. dubius* or *A. camansi* seed protein extracts (1 μg/mL, 3 μg/mL, 6 μg/mL, 11 μg/mL, 46 μg/mL, 92 μg/mL, 184 μg/mL and 367 μg/mL) for 48 h. Afterwards, cells were washed with cell culture media and cytotoxicity measurements were performed in a spectrophotometer at 490 nm using a CellTiter 96^®^ aqueous assay. Nontoxic concentrations were selected for further experiments.

### 3.12. Neuroprotective Evaluation of Protein Extracts

For this experiment, Aβ_1–40_ was dissolved in sterile filtered H_2_O at 235.9 μM. A cytotoxicity test of Aβ_1–40_ on SH-SY5Y was performed to determine the optimal Aβ_1–40_ concentration for the assay and 100 µM Aβ_1–40_ was selected. These 100 μM Aβ_1–40_ samples were incubated at 37 °C with or without nontoxic concentrations of the seed protein extracts (6 μg/mL, 11 μg/mL and 46 μg/mL) for 24 h. After this incubation period, these solutions were added to SH-SY5Y cells (12,500 cell/well) that were allowed to adhere for 24 h and incubated for 48 h at 37 °C. Subsequently, cell viability was determined at 490 nm using a CellTiter 96^®^ aqueous assay.

### 3.13. Statistical Analysis

Statistical analyses were conducted using Student’s *t*-test with two-tailed distributions (unequal variances). Differences were considered significant at *p* < 0.05. All experiments were performed in triplicate.

## 4. Conclusions

Our work demonstrated that *A. dubius* and *A. camansi* seed protein extracts exhibited chaperone activity and consequently were able to inhibit Aβ_1–40_ fibril formation as demonstrated by DLS and ThT measurements. The *A. dubius* sample demonstrated a higher efficacy in inhibiting fibril formation than the *A. camansi* protein extract. This result may be attributed to its higher chaperone activity at the concentration studied. Furthermore, the enzymatic hydrolysis study using alcalase confirmed that the proteins present in the samples played a key role in the inhibition of beta amyloid fibrillogenesis. At nontoxic concentrations (low concentrations), both protein extracts were able to protect SH-SY5Y cells against Aβ_1–40_-induced cytotoxicity. Taking into consideration that current treatments for AD induce severe side effects and only work to alleviate the psychological and behavioral symptoms associated with this disease, the protein extracts from *A. dubius* and *A. camansi* are promising candidates for AD treatment, especially considering that this approach may induce minimal side effects. Overall, our results proved the potential of these protein extracts as novel therapeutics for treating one of the hallmarks of Alzheimer’s disease, beta amyloid fibrillogenesis. This is the first time that the inhibitory capacity and neuroprotective effects of *A. dubius* and *A. camansi* seed protein extracts have been demonstrated. Future work will be focused on the fractionation of both protein extracts and exploring the efficacy of these fractions against Aβ-induced cytotoxicity. Additionally, the encapsulation of the protein extracts in drug delivery systems will be performed. These platforms will be further modified with ligands to target the brain.

## Figures and Tables

**Table 1:** Protein secondary structure content obtained using the server BeStSel.

Conformation	*A. dubius* (%)	*A. camansi* (%)
α-Helix	5.9	0.0
β-Sheet	25.6	36.8
β-Turn	13.5	12.4
Others	54.9	50.7

**Table 2:** Scientific names, local names and parts used of the selected plants.

Scientific Names	Local Names	Parts Used
*Amaranthus dubius* Mart. ex Thell	Red Spinach, Pig’s weed, Bledo	Seeds
*Artocarpus camansi* Blanco	Breadnut, Pana de Pepita	Seeds

## Data Availability

Not applicable.
